# “Not just another Wii training”: a graded Wii protocol to increase physical fitness in adolescent girls with probable developmental coordination disorder-a pilot study

**DOI:** 10.1186/s12887-018-1029-7

**Published:** 2018-02-22

**Authors:** Emmanuel Bonney, Eugene Rameckers, Gillian Ferguson, Bouwien Smits-Engelsman

**Affiliations:** 1 Department of Health & Rehabilitation Sciences, Faculty of Health Sciences, University of Cape Town, Cape Town, South Africa; 2 Department of Physiotherapy, School of Biomedical & Allied Health Sciences, University of Ghana, Accra, Ghana; 3 Adelante Centre of Expertise in Rehabilitation and Audiology, Hoensbroek, the Netherlands; 4 Department of Functioning and Rehabilitation, Maastricht University, Maastricht, the Netherlands

**Keywords:** Active video games, Graded Wii protocol, Physical fitness, Probable DCD, Adolescents

## Abstract

**Background:**

Adolescents with low motor competence participate less in physical activity and tend to exhibit decreased physical fitness compared to their peers with high motor competence. It is therefore essential to identify new methods of enhancing physical fitness in this population. Active video games (AVG) have been shown to improve motor performance, yet investigations of its impact on physical fitness are limited. The objective of this study was to examine the impact of the graded Wii protocol in adolescent girls with probable Developmental Coordination Disorder (p-DCD).

**Methods:**

A single-group pre-post design was conducted to assess the impact of a newly developed Wii protocol in adolescent girls attending school in a low income community of Cape Town, South Africa. Sixteen participants (aged 13-16 years) with p-DCD (≤16th percentile on the MABC-2 test) were recruited. Participants received 45 min Wii training for 14 weeks. Outcome measures included the six-minute walk distance and repeated sprint ability. Information on heart rate, enjoyment and perceived exertion ratings were also collected.

**Results:**

Significant improvements in aerobic and anaerobic fitness were observed. The participants reported high enjoyment scores and low perceived exertion ratings. The graded Wii protocol was easily adaptable and required little resources (space, equipment and expertise) to administer.

**Conclusions:**

The findings provide preliminary evidence to support the use of the graded Wii protocol for promoting physical fitness in adolescent girls with p-DCD. Further studies are needed to confirm these results and to validate the clinical efficacy of the protocol in a larger sample with a more robust design.

## Background

Developmental Coordination Disorder (DCD) is a neurodevelopmental condition that impairs the development of motor skills and coordination [1]. Children with DCD experience difficulty with motor tasks and participate less in physical activity. The symptoms of DCD track from childhood into adolescence [2, 3]. Compared to their typically developing peers, children and adolescents with DCD exhibit low motor competence and decreased physical fitness, and tend to have greater risk for overweight and obesity [4]. Given that children with DCD experience increased risk of developing cardiovascular diseases [4], fitness promotion may be a vital preventative strategy for mitigating adverse health complications. Although the linkage between physical fitness and motor competence is reported to be stronger in adolescence [5], physical fitness declines from childhood to adolescence [6, 7]. Therefore, it is critical to identify new ways of boosting physical fitness among adolescent populations with motor coordination problems.

Lately, the use of active video games in neuromotor rehabilitation is increasingly becoming pervasive. Active video games (AVGs) are motion-controlled computer games used to promote physical activity [8]. The Nintendo Wii, used in the present study, consists of a video-based console, handheld remote and balance board that allow the player to interact with the virtual environment via wireless controller. Players use whole body movements (mostly weight shifting in different directions) and arm gestures to control the game. To enhance the players’ performance, the Wii provides several augmented feedback (visual and auditory forms) before, during and at the end of each episode of play [9, 10]. Earlier studies have shown that the Wii elicits improvements in motor coordination and aspects of physical fitness in young children. Smits-Engelsman et al. [11] evaluated the effectiveness of the Wii in children with DCD and their typically developing peers (TD). After 5 weeks, both groups improved on functional strength and anaerobic fitness. This suggests that the Wii might be a useful tool to enhance physical fitness in individuals with low motor competence. In another study, the authors investigated the effects of the Wii on motor and psychological outcomes in children [12]. The children demonstrated improvements in motor proficiency and emotional well-being. In contrast, a recent study revealed that the Wii offers lesser benefits in motor proficiency, cardiorespiratory fitness and functional strength [13]. Also, it has been established that the Wii can be implemented as an adjunct for treating children with developmental delay [14] and those with motor coordination deficits [15]. There is growing evidence to support the use of the Wii for balance control training in children and adults with motor problems [16, 17]. Though active video games have been found to increase total body movement in adolescents [18], the impact of these games on physical fitness in adolescents with DCD has not been determined.

Providing opportunities for physical activity in adolescent girls with insufficient opportunity (low income community dwellers) [13] is increasingly becoming difficult. Two main reasons have been provided for this challenge. First, traditional physical activities are viewed as physically demanding and are therefore undesirable for this population. Additionally, engaging in outdoor activities and sports do not seem appealing due to safety concerns and lack of resources in most low income settings. Secondly, girls with motor problems tend to exhibit motor impairments that hinder their participation in everyday tasks. In South Africa, girls are reported to have high prevalence of overweight and obesity compared to boys. This problem has been partly attributed to low motor competence [19]. Also, it is well established that during adolescence, several unhealthy habits become entrenched [20], with negative implications for adult life. Given the significant influence of physical fitness on health outcomes, developing new interventions that can be implemented to increase physical fitness in adolescent populations with DCD is reasonable. Components of physical fitness such as cardiovascular endurance, muscular strength, and anaerobic performance are compromised in individuals with motor problems [21, 22] leading to reduced perceived motor competence and withdrawal from physical activity [23]. As motor problems trail from childhood into adolescence, adolescents with low motor competence may struggle with daily activities, academic work and social roles. Consequently, their overall health status may deteriorate if tailor-made interventions are not provided.

Based on earlier findings which sought to suggest that the Wii might improve physical fitness in children with DCD [11, 13], this study was set up as an initial step to inform a larger randomized controlled trial aimed at evaluating the effectiveness of a newly developed Wii intervention (the graded Wii protocol). Therefore, the primary purpose of the study was to examine the impact of the graded Wii protocol in adolescent girls with probable DCD. Specifically, we investigated the effects of the graded Wii protocol on aerobic and anaerobic fitness. To accomplish this, the following were assessed;

1. changes in performance on field-based aerobic and anaerobic fitness tests
2. experiences of adolescent girls during the training sessions
3. exercise intensity during the training sessions
4. the ease of implementation of the protocol as reported by the supervising therapists and
5. injury occurrence during the training sessions.

## Methods

### Design

The study was a single group pre-post design. In South Africa, the prevalence of overweight and obesity is higher in females than males, especially among those living in low income communities [24]. Compared to boys, girls exhibit low motor competence more often [19]. For this reason, 16 girls aged 13-16 years, attending a local school in a low income community of Cape Town, South Africa, were recruited. The school serves underprivileged black communities and is primarily attended by children of black South Africans (100%) who share similar socioeconomic status. Parents and participants gave written informed consent before involvement. The informed consent process varied according to age. Essentially, the content of the consent forms used was somewhat similar for both the parents and children. But the written expression and structure were aligned to the children’s cognitive abilities to facilitate comprehension. Inclusion criteria included a score ≤ 16th percentile on the Movement Assessment Battery for Children 2nd edition (MABC-2) test [25] (Criterion A). Participants did not report any medical condition (including cerebral palsy and epilepsy) known to affect motor performance and were at a mainstream high school confirming the absence of intellectual or cognitive impairment (Criterion D). Also, the participants had normal IQ and good or corrected vision. It has been suggested that the term DCD should be used to refer to individuals with motor coordination problems that satisfy all the diagnostic criteria stipulated in the Diagnostic and Statistical Manual of Mental Disorders, Fifth Edition (DSM-V) [26–28]. In this study, our sampled participants exhibited motor coordination deficits, but we could not confirm all the DSM-V diagnostic criteria and so we decided to refer to them as having probable DCD (p-DCD) [29, 30].

Ethical approval for the study was granted by the Human Research Ethics Committee of the University of Cape Town (HREC REF: 232/2016) and permission was obtained from the school’s principal. The estimated sample size was determined using previous data [31]. Based on this information, it was established that 16 participants were needed to detect a difference between pre and post training measures with power of 0.8 and effect size of 0.7. Outcome measures were assessed at baseline and at the end of the training period. None of the participants had prior Wii experience and no participant played any of the Wii games outside the training hours.

### Intervention

The graded Wii protocol was developed from commercially available Wii games selected from the Nintendo Wii system. The protocol was created by qualified physiotherapists with experience in exergames rehabilitation. The protocol incorporated the Newell’s constraints theory [32, 33] and exercise progression principles [34, 35]. Specifically, the Wii games that had the tendency to stimulate the cardiovascular system for positive benefits in strength and conditioning were selected by two experienced independent assessors. A third person also re-evaluated all the selected games and developed the protocol (the graded Wii) to consist of various combinations of games and their adaptations. Two main criteria were adopted for game selection and evaluation; (1) games should require whole bodily movement to control the avatar (2) games should be amenable to progressive external modifications without limiting playability. Backpacks with sandbags (which weighed 1 kg & 3 kg) and wooden platforms (25 cm high) were used to externally change the physical demands of the games. These items were used to progressively increase the level of challenge and physiological load over the training period. Each participant was required to play 8 games for 45 min per session, once weekly for 14 weeks. For each training session, the participants were required to play different variation of games chosen from the 4 available game categories (aerobics, balance, muscle workout and yoga). A detailed scheme of the protocol is provided in Table 4 in Appendix. During Weeks 1 to 5, the participants were instructed to familiarize themselves with the selected games; hence no alterations were introduced throughout this period. From Week 6 to 14, gameplay was gradually adjusted to increase the physiological load. This was done through the use of backpacks filled with weights (1 kg at the midpoint and 3 kg towards the end of the training period) and a 25 cm high wooden platform. The training was delivered to a maximum of six participants simultaneously in an enclosed room. Six Wii consoles and TVs were arranged and partitioned so that participants were not distracted by other players. Each session was supervised by physical therapy and human movement science students.

Prior to each session, participants received brief orientation of the Wii games. The supervisors used the orientation period to introduce the games for the session and to encourage the participants to fully engage with the protocol to gain positive benefits in physical fitness. Also, the orientation segment afforded the participants unique opportunity to ask questions regarding aspects of the protocol that were unclear and to report any technical difficulties with the set up.

### Measurements

Demographic data including age, grade and hand preference were collected from each participant. Also, BMI and physical activity data (number of days in which participants were physically active for 30 min or more) were collected. Assessments were done in the school’s playground by two groups of independent assessors at pre and post intervention. The second group of assessors was blinded to the pretest scores. Participants’ perceived exertion, heart rate and enjoyment ratings were monitored during the training and at the end of each session. Injuries that occurred during the training were also recorded. Each supervisor was interviewed to share his or her experiences regarding the organization of the protocol.

### Physical fitness, heart rate, perceived exertion, enjoyment and experiences of supervisors

#### Physical fitness

The six-minute walk test (6MWT) was used to evaluate the aerobic fitness of the participants. The test was chosen because it uses everyday functional activity (walking), and has been extensively used in studies involving children and adolescents. Also, it is known to be safe, easy to perform and highly acceptable to children [36]. It provides a valid and inexpensive means to measure functional capacity in children [37–39]. The 6MWT measures aerobic fitness across all ages. The test was executed according to recommended protocol [36] over a 20 m distance walkway. During the test, each participant was instructed to cover much distance in 6 min. However, they were allowed to rest if they wished and continued when they were ready to do so [37–39]. Two trials were performed on the same day with a 30-min rest between trials and the mean score is reported in this paper. Test-retest reliability of the 6MWT is high [ICC 0.94 (95% CI = 0.89–0.96)] in healthy children indicating high reliability, and the Smallest Detectable Difference (SDD) is estimated to be 50 m [36].

In addition, the Muscle Power Sprint Test (MPST) was used to assess anaerobic fitness. The MPST involved the completion of six 15 m sprints at maximum speed with 10 s rest interval. The test took place on a 15 m level ground at the school’s soccer field. Each participant’s sprint time was recorded using stopwatches in milliseconds [11]. Based on the time and weight of the participant, the mean power (Watts) over 6 repetitions was calculated. Greater mean power indicates the ability to maintain power output over time and translates into better maintenance of anaerobic performance. The mean power of the MPST demonstrated an ICC of 0.90 (95% CI = 0.85-0.99) for test-retest reliability in this age group [40]. Steenman and colleagues [40] showed with a Bland-Altman plot that there was no significant learning effect between the first and second trials. In the same paper, the measurement error was found to be 16.8 W with an estimated SDD of 33 W.

#### Heart rate

The American College of Sports Medicine recommends that individuals with chronic diseases and disabilities achieve moderate intensity physical activity (40-70% of maximal HR) for improved cardiorespiratory fitness [41]. To monitor exercise intensity during the training sessions, participants wore Polar heart rate monitors (Polar S810) across their chest accompanied by wristwatches. The Polar S810 has good accuracy compared to ambulatory [42] and supine ECG [43]. Participants’ resting heart rate (HR) and peak heart rate were recorded. Resting heart rate was recorded in sitting (3-5 min) whereas peak heart rate was recorded in the course of play. Estimated maximum heart rate based on resting HR and participants’ age was also calculated using the formula derived by Gulati [44]: Estimated maximum Heart rate (HR_max_) = 206 − (0.88 × age).

Lastly, we calculated the percentage of the estimated HR reached during the training to check if individual peak HR was above the recommended level.

#### Perceived exertion

Table 1 shows the Borg’s Rating of Perceived Exertion (RPE) scale that was used to measure the participants’ perceived exertion. The scale consists of numerical values (6-20, where 6 means “no exertion at all” and 20 means “maximal exertion”), and expresses one’s subjective feeling regarding the intensity of an exercise programme. The tool is reported to be valid and reliable [45].

**Table 1:** Borg’s Rate of Perceived Exertion (RPE) Scale

Rate of Perceived Exertion (RPE) Scale	
6	
7	Very very light
8	
9	Very light
10	
11	Fairly light
12	
13	Somewhat hard
14	
15	Hard
16	
17	Very hard
18	
19	Very very hard
20	Maximum exertion

#### Enjoyment rating scale

Since enjoyment is an important motivator, the Enjoyment rating scale, was used to measure the participants’ enjoyment experienced during the training sessions. The scale uses 5 smiley faces with numeric scores (0-4, 0 means boring; 4 is awesome) to assess how much the participants enjoy playing the Wii games at any given time. The Enjoyment rating scale used in this study has been adequately described elsewhere [16]. It was hypothesized that the harder the level of challenge, the less enjoyable participants would find the games.

#### The supervisors’ experiences

At the end of the training period, each supervisor was requested to share their experiences regarding the organization and delivery of the protocol. Also, they were asked to report on technical difficulties associated with the administration of the protocol. Additionally, injuries that occurred during the training sessions were monitored and recorded.

### Data analysis

Data were checked for normality using the Kolmogorov-Smirnov test and appropriate analyses are reported. Mean and standard deviation (SD) are reported for age, height, weight, and BMI, and pretest values on the MABC-2 test. To estimate the intensity of the training, averages of the RPE, and peak HR over 14 sessions are reported. Also, enjoyment over the 14 sessions was assessed. The individual Peak HR was compared to the percentage of the estimated maximum HR. Next, correlation between Peak HR and RPE and between Peak HR and enjoyment scores was determined to ascertain if greater exertion made playing the games less fun. Also, we tested if the aerobic fitness (six-minute walk distance) changed between pre and posttest using a paired *t*-test. To test if anaerobic fitness and susceptibility to fatigue changed, the 6 runs of the 15 m sprint test were analyzed using a repeated measure ANOVA with runs (6 repetitions) and time of measurement (pre post) as within subject factors at *p* < 0.05. Since fatigue index or the percentage decrement score is believed to be a valid indicator of anaerobic capacity, we also calculated the percentage decrement score using the recommended formula [46]. The percentage decrement score quantifies fatigue by comparing actual performance to an imagined ‘ideal performance’.

Next we calculated single-group, pretest–posttest raw score effect size [47]; A standardized mean difference was calculated by subtracting the mean of the scores at posttest from the mean at pretest and dividing this raw mean difference by the standard deviation of the scores at the first time point. The magnitude of the effect size was interpreted using the conventions of Cohen: small = 0.2, medium = 0.5 and large = 0.8 [48]. To compensate for test-retest bias, we looked at the individual change and reported the number of children that improved more than the SDD on the 6MWT and MPST. All statistical analyses were performed with SPSS (SPSS Inc., version 23).

## Results

### Baseline characteristics of participants

The mean age of the participants was (14.5 ± 1.0 years, range 13-16 years). The mean weight and BMI was (68.1 ± 18.5 kg) and (27.5 ± 7.3 kg/m^2^) respectively. Eleven were classified as “at risk of DCD” and five had “definite motor impairments” on the MABC-2 test (Mean TSS ± SD: 62.8 ± 5.6; Range: 48-69) [25]. The median reported days that the participants were physically active for 30 min or more was 3. Only 3 out of the 16 reported to be active for 30 min every day. All the participants scored below the 5th percentile on the 6MWT (mean walking speed 1.13 m/s ± 0.19) [49]. Nine perceived themselves as being low motor skilled and all reported their willingness to be more active.

### Participants’ characteristics during training sessions

**Fig. 1:**
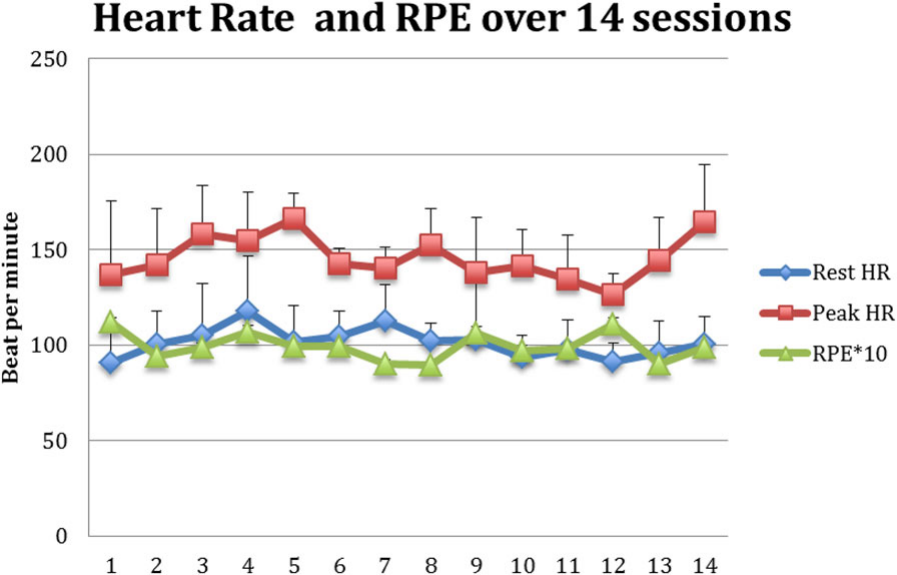
Participants’ resting HR, Peak HR and perceived exertion (RPE × 10) during the 14 sessions. Note: Error bars indicate Standard Error

**Fig. 2:**
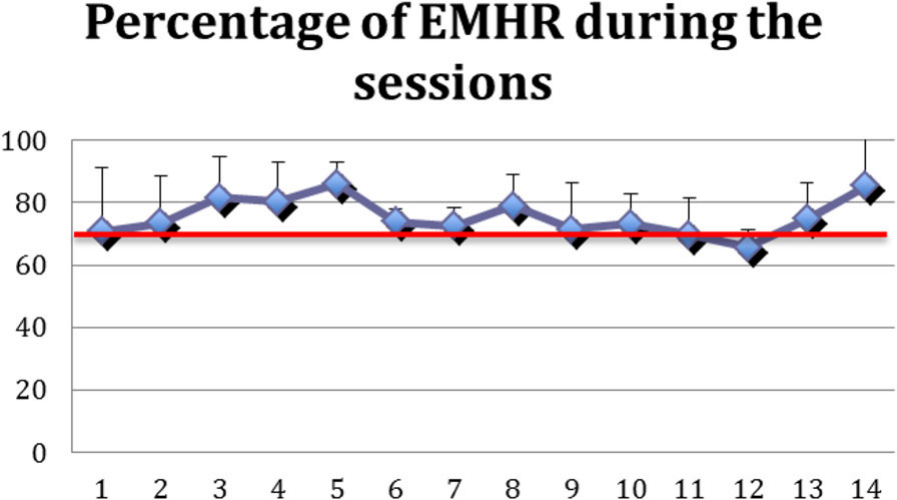
Percentage of the estimated maximum heart rate (EMHR) reached across 14 sessions. Note: Error bars indicate Standard Error and Red line represents target EMHR of 60%

As shown in Fig. 1, the average peak HR was (148.1 ± 23.4) beats per minute (bpm) and the mean increase in HR per training computed from the difference in resting HR and peak HR values was 48.3 ± 24.6 bpm. The estimated max HR was 193.3 ± 0.78 bpm. The measured mean peak HR over all sessions reached 74.9% (SD: 13.1) of the estimated max HR (Fig. 2). Of all the HR readings, 88.1% were above the 60% level and 61.9% above the 70% level. This confirms that in most cases an adequate maximum level of intensity was reached.

**Fig. 3:**
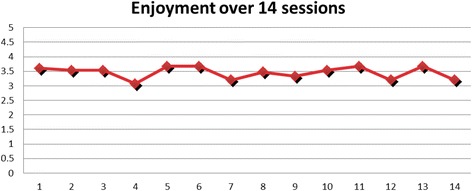
Enjoyment of games played by participants over 14 sessions

Overall, the participants liked the training (Fig. 3). The mean enjoyment score was 3.5 ± 0.75 (Median: 4). 58.6% rated the training as awesome, 30.5% as fun, 8.6% as a bit of fun and 2.4% as boring. Interestingly, there was no correlation between the peak HR and enjoyment scores.

The mean RPE was 9.93 ± 2.85 (Median: 9). 46.2% of all the ratings were at least 11 or more whereas 8.6% reported 13 or more. Because of the skewed distribution of the enjoyment scores, we tabulated the percentage of choices of the enjoyment scale against the RPE ratings. It can be noticed that low and high intensity ratings could either be felt as awesome or boring (Table 2).

**Table 2:** Values for ratings of perceived exertion (RPE) and enjoyment scale

RPE	6	7	8	9	10	11	12	13	15	16	17	
Enjoyment- boring	0	0	0	0	0	1	0	3	0	0	1	5
Enjoyment- a bit of fun	0	1	1	3	0	3	0	6	2	0	2	18
Enjoyment- Fun	11	9	2	9	3	15	2	8	4	0	1	64
Enjoyment- Awesome	14	24	3	31	2	29	0	12	5	1	2	123
Total	25	34	6	43	5	48	2	29	11	1	6	210

*Abbreviation*: *RPE* ratings of perceived exertion

No correlation was found between Peak HR and the RPE. Low non-parametric correlations (r_s_ = 0.12, *p* = 0.008) were seen between the increase in HR during the training and RPE.

### Comparison of physical fitness outcomes (pre and post)

After the training, the recorded six-minute walk distance (6MWD) was longer (≥20%) in both trials (6MWD1; pre 409 ± 66.9 m, post 481 ± 63.0 m, t = − 3.26, *p* = 0.005, d = 1.11; 6MWD2; pre 401 ± 65.0, post 509 ± 34.0, t = − 5.18, *p* < 0.001, d = 2.08). Respiratory rate (RR) in the posttest increased (t = − 5.88, p < 0.001) compared to the pretest during the first trial of the 6MWT. No differences in HR (*p* = 0.167 and *p* = 0.736) or RPE (*p* = 0.089 and *p* = 0.743) between pre and posttest was found for both test occasions (For means see Table 3). The test was not terminated prematurely for any participant.

**Table 3:** Pre and post mean scores of outcomes

Variables	Pre (Mean ± SD)	Post (Mean ± SD)	t, or F value (df = 15)	*P*-value
Six minute walk distance trial 1(m)	409 ± 66.9	481 ± 63.0	−3.26	0.005
Respiratory rate (breaths per minute)	92 ± 12.9	126.1 ± 21.2	−5.88	0.001
Heart rate (bpm)	123.7 ± 17.6	133.5 ± 22.1	−1.45	0.167
Rate of Perceived Exertion (#)	9 ± 2.6	10.2 ± 2.2	−1.82	0.089
Six minute walk distance trial 2 (m)	401 ± 65.0	509 ± 34.0	−5.18	0.001
Respiratory rate (breaths per minute)	94.8 ± 13.4	99.8 ± 31.9	−0.58	0.569
Heart rate (bpm)	126.8 ± 21.6	129.3 ± 18.7	−0.34	0.736
Rate of Perceived Exertion (#)	8.4 ± 2.3	8.7 ± 2.2	−0.33	0.743
Mean 15-m sprint time (s)	4.32 ± 0.68	3.89 ± 0.47	4.56	0.005
Mean power (Watts)	221.2 ± 101.9	341.3 ± 166.7	−2.69	0.017

*Abbreviations*: *m* metre, *#* number, *s* seconds, *bpm* beats per minute

**Fig. 4:**
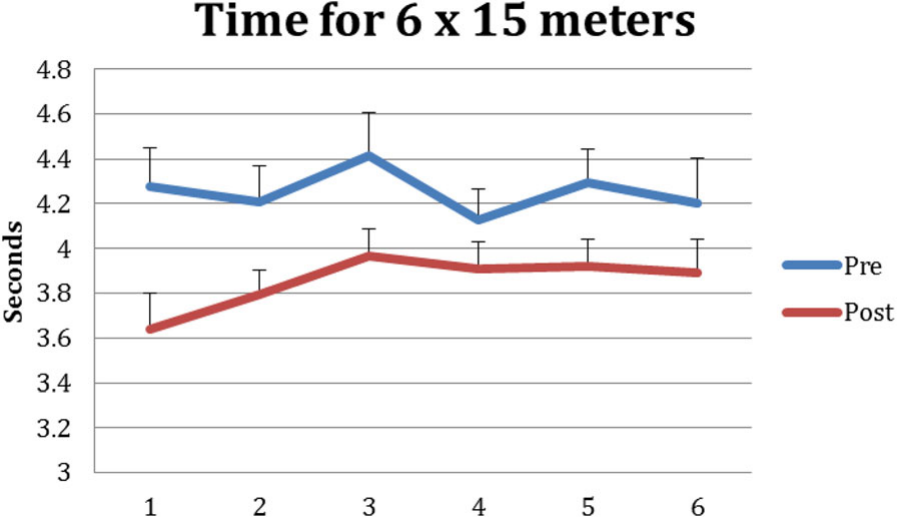
Running time before (pre) and after (post) training for the 6 repetitions of the 15 m sprint. Note: Error bars indicate Standard Error

The 15 m sprint time decreased by 10%; from (4.32 ± 0.68 s) to (3.89 ± 0.47 s) (F (1, 15) 4.56, *p* = 0.05, η^2^ = 0.23) (Fig. 4). No main effect of repetition was found, indicating that repeated sprints did not lead to poorer (or better) performance. The interaction effect with number of sprints and time of testing was also not significant. Moreover, no significant difference was found in the percentage decrement score between pre and post test (Mean 15.67 ± 9.58 and 18.67 ± 17.2 for pre and post, respectively; t (1, 15)-0.60; *p* = 0.56). Generally, the participants did not slow down much upon repeated trials and this was similar in pre and posttest (Fig. 4).

### Individual change

Of the 16 children, 11 improved more than the SDD of the 6MWT whereas 12 improved more than SDD of the Mean Power produced from the MPST data.

### Experiences of supervisors

Regarding the training supervisors’ experience, all reported that when equipment is available, it is simple to administer the graded Wii protocol. They also revealed that it required little space and minimal technical expertise. The supervisors suggested that for the training to be effective, it is important to explain the aim of a gaming session, and to establish good rapport with the participants. Provision of positive verbal feedback (Knowledge of Performance) was also highlighted as critical for successful performance. Lastly, no injury was recorded during the training.

## Discussion

This pilot study was designed to examine the impact of the graded Wii protocol on aspects of physical fitness in adolescent girls with p-DCD. While the usefulness of AVGs has been demonstrated in children with DCD, its impact on physical fitness remains unknown. The study involved a sample of physically unfit girls with low motor competence. Besides, the girls had limited opportunities to participate in physical activity. This could be due to cultural and environmental challenges such as lack of facilities, poor weather conditions and unsafe neighborhoods.

Generally, we have demonstrated that the collective experience of the girls during the training sessions was positive (fun to awesome) and that they reached the required 60-70% estimated peak HR. More importantly, there were significant improvements in walking distance and sprint time, an indication of increased physical fitness. Additionally, the graded Wii protocol was easy to administer even with little resources. This suggests that the Wii protocol might probably be useful for promoting fitness in situations where it is impossible or unsafe for people to engage in outdoor activities or sports. Given the fact that no control group was used in our design, other explanations for the observed changes cannot be ruled out, one being test-retest effects. However, the tests used have high test-retest reliability; the reported effect sizes are moderate to large. To our knowledge, there is only one intervention study that has used the 6MWT and has reported effect sizes of a non-treatment control group [29]. The reported effect size of 0.12 in that study is much smaller than the 1.11 and 2.08 in the present study. Moreover, most children improved beyond the Smallest Detectable Change. Nevertheless, we cannot exclude other explanations for the observed changes. Therefore, further investigations with control groups are required to confirm the outcomes of the present study. Indeed, if a protocol of this nature could elicit individual changes in aerobic and anaerobic fitness, then it could be considered as a viable alternative for physical education programmes in schools where physical educators are in short supply. The protocol can also be implemented in less-endowed communities to promote physical activity and fitness, as fitness programmes are often not available in such settings.

Although the graded Wii protocol was adjudged entertaining and enjoyable, it created sufficient challenge for improved outcomes among the participants. This suggests that the Wii games could be manipulated to provide adequate intensity for health benefits, without reducing the players’ motivation and enjoyment. The introduction of add-ons (such as backpacks with weights) produced competitive stimulus and increased the participants desire to succeed and might explain the observed changes in HR. While the RPE was low for the participants, their peak HR was higher than the required estimated peak HR. Importantly, exercise intensity was considered adequate enough to improve the physical fitness indicators assessed in the present study.

The perception of exertion was low for a greater proportion of the girls. Robert et al. [41] reported much higher perceived exertion ratings among children with cerebral palsy. This disparity could be attributed to the differences in the nature of games, level of motor impairments and level of maturity (differences in age). In that study, younger children (7-12 years) played only jogging and bicycling games. These two games exert the cardiorespiratory systems and given that children with cerebral palsy have reduced cardiorespiratory fitness, we expect their perception of exertion to be much higher than our sample that played a mix of aerobics, balance, strength and yoga games.

The exercise intensity was relatively high and elicited significant improvements in both aerobic and anaerobic fitness. This finding does not conform to previous reports by Nitz et al. [9]. In their study, cardiovascular endurance did not yield any improvements in women (aged 30-58 years) who had two 30 min training per week for 10 weeks. Several reasons could explain this discrepancy. Firstly, our participants are much younger and had lower levels of motor coordination, physical activity and fitness. Also, the intensity of the protocol (a product of time, frequency and game difficulty) was higher than what was reported. In the present study, extra loads were progressively added to increase the physiological load of the games. These loads (backpacks) provided some kind of resistance and increased the strength of the muscles of the legs. The wooden blocks elevated the balance board and eventually raised the participants’ base of support. Thus, increasing the task constraints regarding their step-up pattern and balance control.

Though this study provides preliminary evidence to support the adaptation of the Wii games to increase measures of physical fitness, there are several limitations that should be recognized. The major limitation of this study is the lack of a control group. The lack of a control group makes the present study vulnerable to threats of internal validity. It was practically impossible to include a control group due to the insufficient number of participants and other ethical concerns. We recommend that future studies should consider the inclusion of controls when assessing the effects of the graded Wii protocol in a much larger sample. Another limitation is the use of peak HR as indicative of training intensity. Mean HR and time above 60-70% max HR would be more appropriate indicators of training intensity. In the present study we could not record HR continuously over an entire training session. It would be useful to employ a more appropriate measure to estimate the training intensity in future research. Given that it has been shown that intervention works in children with DCD [50] it could be unethical to have a non-treatment control group, and therefore a cross-over design might be valuable. Research on the effects of the graded Wii protocol on age and gender should be considered in future works. Also, investigations of the impact of the graded Wii protocol in individuals with and without co-occurring disorders and in populations with neurodevelopmental disorders such as Cerebral palsy, intellectual disabilities and Autism Spectrum Disorder is recommended. Studies that would increase the training frequency to 2 or 3 times per week may yield greater outcomes. Lastly, the impact of the graded Wii protocol on activity levels, motor skills and perceived competence might be worth considering.

## Conclusions

Based on the findings of this study, it can be concluded that the graded Wii protocol could be implemented to increase important components of physical fitness in adolescent girls with probable DCD. Since the participants found the games enjoyable even in the midst of all the adaptations, the protocol could be easily used to stimulate physical activity and to promote fitness in sedentary individuals who have little or reduced motivation to exercise.

## Appendix

**Table 4:** Details of the graded Wii protocol administered over 14 sessions

Week #	Names of the selected games	Number of repetitions per session	Game adaptations
1	Jogging (short distance)	2	Familiarization phase. No add-on
	Single leg extension	10	
	Lunge	1	
	Hula hoop	1	
	Soccer heading	1	
	Ski slalom	1	
	Warrior	1	
	Half moon	1	
2	Jogging (short distance)	2	Familiarization phase. No add-on
	Hula hoop	2	
	Rowing squat	1	
	Torso twist	1	
	Penguin slide	1	
	Perfect 10	1	
	Obstacle course	1	
	Sun salutation	1	
3	Jogging (short distance)	2	Familiarization phase. No add on
	Rhythm cycling	2	
	Single leg twists	10	
	Jack knife	1	
	Boxing	1	
	Penguin slide	1	
	Table tilt	1	
	Warrior	1	
4	Jogging (short distance)	2	Familiarization phase. No add on
	Basic steps	2	
	Lunge	1	
	Rowing squat	10	
	Boxing	1	
	Soccer heading	1	
	Penguin slide	1	
	Half moon	1	
5	Jogging (short distance)	2	Familiarization phase. No add on
	Island cycling Short	2	
	Basic steps	10	
	Single leg extension	1	
	Torso twists	1	
	Ski slalom	1	
	Perfect 10	1	
	Sun salutation	1	
6	Jogging (Long distance)	2	The games were graded by making participants carry a backpack with 1 kg load while gaming. The height of the balance board remained unchanged.
	Hula hoop	2	
	Rowing squat	2	
	Single leg twist	10	
	Obstacle course	2	
	Penguin slide	2	
	Half moon	2	
	Tree	2	
7	Jogging (Long distance)	2	The games were graded by making participants carry a backpack with 1 kg load while gaming. The height of the balance board remained unchanged.
	Basic steps	2	
	Kung Fu	2	
	Single leg extension	10	
	Table tilt	2	
	Soccer heading	2	
	Warrior	2	
	Half moon	2	
8	Jogging (Long distance)	2	The games were graded by making participants carry a backpack with 1 kg load while gaming. The height of the balance board remained unchanged.
	Basic steps	2	
	Rowing squat	2	
	Single leg twist	10	
	Penguin slide	2	
	Obstacle course	2	
	Tree	2	
	Palm tree	2	
9	Jogging (Long distance)	2	The games were graded by making participants carry a backpack with 1 kg load while gaming. The height of the balance board remained unchanged.
	Hula hoop	2	
	Rhythm boxing	2	
	Single leg extensions	10	
	Lunge	2	
	Sun salutation	2	
	Palm tree	2	
	Just Dance	1 song	
10	Jogging (Long distance)	2	The games were graded by making participants carry a backpack with 1 kg load while gaming. The height of the balance board remained unchanged.
	Hula hoop	2	
	Torso twists	2	
	Table tilt	2	
	Ski slalom	2	
	Sun salutation	2	
	Half moon	2	
	Just Dance	3 songs	
11	Jogging Long	2	The load was increased from 1 kg to 3 kg. Each participant carried the new load (3 kg) during gameplay. Also, the height of the balance board was raised with a 25 cm high elevation (wooden platform).
	Step Basics	2	
	Kung Fu	2	
	Rowing squat	2	
	Single leg extension	20	
	Obstacle course	2	
	Half moon	2	
	Just Dance	5 songs	
12	Jogging (Long distance)	2	The load was increased from 1 kg to 3 kg. Each participant carried the new load (3 kg) during gameplay. Also, the height of the balance board was raised with a 25 cm high elevation (wooden platform).
	Step Basics	2	
	Rhythm boxing	2	
	Rowing squat	20	
	Soccer heading	2	
	Ski slalom	2	
	Half moon	2	
	Just Dance	5 songs	
13	Jogging (Long distance)	2	The load was increased from 1 kg to 3 kg. Each participant carried the new load (3 kg) during gameplay. Also, the height of the balance board was raised with a 25 cm high elevation (wooden platform).
	Basic steps	2	
	Single leg extension	20	
	Lunge	2	
	Penguin slide	2	
	Tree	2	
	Palm tree	2	
	Just Dance	5 songs	
14	Jogging Long	2	The load was increased from 1 kg to 3 kg. Each participant carried the new load (3 kg) during gameplay. Also, the height of the balance board was raised with a 25 cm high elevation (wooden platform).
	Step basics	2	
	Single leg twist	20	
	Table tilt	2	
	Kung Fu	2	
	Sun salutation	2	
	Half moon	2	
	Just Dance	5 songs	

## Abbreviations

6MWD: Six-minute walk distance
6MWT: Six-minute walk test
ANOVA: Analysis of variance
AVG: Active video game
BMI: Body mass index
CI: Confidence interval
DCD: Developmental coordination disorder
DSM-V: Diagnostic and statistical manual of mental disorders, Fifth Edition
ECG: Electrocardiogram
HR: Heart rate
ICC: Intraclass correlation coefficient
IQ: Intelligence quotient
MABC-2: Movement Assessment Battery for Children second edition
MPST: Muscle power sprint test
p-DCD: probable Developmental Coordination Disorder
RPE: Rating of perceived exertion
RR: Respiratory rate
SDD: Smallest detectable difference
TD: Typically developing
TSS: Total standard score
